# *N*′-[1-(4-Nitro­phen­yl)ethyl­idene]acetohydrazide

**DOI:** 10.1107/S1600536808040919

**Published:** 2008-12-10

**Authors:** Yu-Feng Li, Lian-Cai Du, Fang-Fang Jian

**Affiliations:** a Microscale Science Institute, Department of Chemistry and Chemical Engineering, Weifang University, Weifang 261061, People’s Republic of China; b Microscale Science Institute, Bioengineering Institute, Weifang University, Weifang 261061, People’s Republic of China; c Microscale Science Institute, Weifang University, Weifang 261061, People’s Republic of China

## Abstract

The title compound, C_10_H_11_N_3_O_3_, was prepared by the reaction of acetohydrazide and 1-(4-nitro­phen­yl)ethanone. The asymmetric unit contains two crystallographically independent mol­ecules. Inversion-related mol­ecules form dimers, in which two N—H⋯O hydrogen bonds generate an inter­molecular *R*_2_^2^(8) ring.

## Related literature

For possibile analytical applications of Schiff bases, see: Cimerman *et al.* (1997 ▶). For a related structure, see: Girgis (2006 ▶).
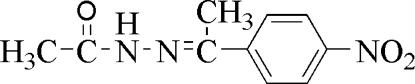


## Experimental

### 

#### Crystal data

C_10_H_11_N_3_O_3_
*M* *_r_* = 221.22
Triclinic, 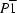

*a* = 8.4453 (17) Å
*b* = 9.5438 (19) Å
*c* = 14.820 (3) Å
α = 72.66 (3)°
β = 77.37 (3)°
γ = 75.59 (3)°
*V* = 1090.7 (4) Å^3^
*Z* = 4
Mo *K*α radiation
μ = 0.10 mm^−1^
*T* = 293 (2) K
0.25 × 0.20 × 0.18 mm

#### Data collection

Bruker SMART CCD area-detector diffractometer
Absorption correction: none
6035 measured reflections
4037 independent reflections
2570 reflections with *I* > 2σ(*I*)
*R*_int_ = 0.027

#### Refinement

*R*[*F* ^2^ > 2σ(*F* ^2^)] = 0.052
*wR*(*F* ^2^) = 0.159
*S* = 1.04
4037 reflections
297 parameters
H atoms treated by a mixture of independent and constrained refinement
Δρ_max_ = 0.22 e Å^−3^
Δρ_min_ = −0.21 e Å^−3^

### 

Data collection: *SMART* (Bruker, 1997 ▶); cell refinement: *SAINT* (Bruker, 1997 ▶); data reduction: *SAINT*; program(s) used to solve structure: *SHELXS97* (Sheldrick, 2008 ▶); program(s) used to refine structure: *SHELXL97* (Sheldrick, 2008 ▶); molecular graphics: *SHELXTL* (Sheldrick, 2008 ▶); software used to prepare material for publication: *SHELXTL*.

## Supplementary Material

Crystal structure: contains datablocks global, I. DOI: 10.1107/S1600536808040919/at2663sup1.cif

Structure factors: contains datablocks I. DOI: 10.1107/S1600536808040919/at2663Isup2.hkl

Additional supplementary materials:  crystallographic information; 3D view; checkCIF report

## Figures and Tables

**Table 1:** Hydrogen-bond geometry (Å, °)

*D*—H⋯*A*	*D*—H	H⋯*A*	*D*⋯*A*	*D*—H⋯*A*
N1—H1*A*⋯O1^i^	0.84 (3)	2.16 (3)	2.989 (3)	170 (2)
N4—H4*A*⋯O4^ii^	0.88 (3)	2.08 (3)	2.952 (3)	172 (2)
C4—H4*B*⋯N1	0.96	2.42	2.826 (3)	105
C4—H4*B*⋯O1^i^	0.96	2.28	3.243 (3)	175
C10—H10*A*⋯O6^iii^	0.93	2.49	3.216 (3)	135
C14—H14*A*⋯N4	0.96	2.41	2.821 (3)	105
C14—H14*A*⋯O4^ii^	0.96	2.36	3.317 (3)	173
C16—H16*A*⋯O3^iv^	0.93	2.50	3.388 (4)	159
C20—H20*A*⋯N5	0.93	2.43	2.740 (3)	100

Symmetry codes: (i) 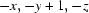; (ii) 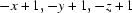; (iii) 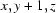; (iv) 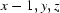.

**Fig. 1.**
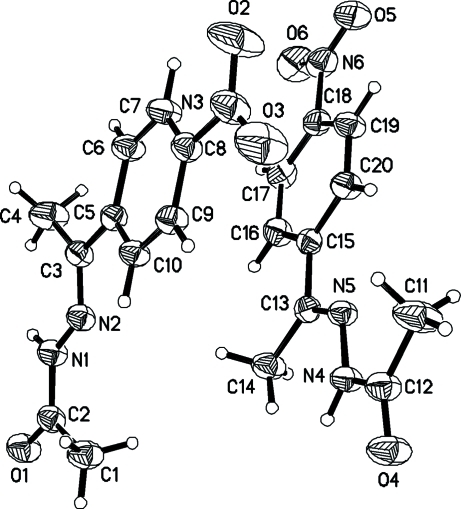
The structure of the title compound showing 30% probability displacement ellipsoids and the atom-numbering scheme.

## supplementary crystallographic information

### Comment

Schiff bases have received considerable attention in the literature. They are attractive from several points of view, such as the possibility of analytical application (Cimerman, *et al.*, 1997). As part of our search for new schiff base compounds we synthesized the title compound (I), and describe its structure here.

As shown in Fig. 1, the asymmetric unit contains two crystallographically independent molecules. The C3—N2 bond length of 1.277 (3) Å and C13—N5 bond length of 1.287 (3) Å is comparable with C—N double bond [1.281 (2) Å] reported (Girgis, 2006).

In the structure, there exist C-H···O, C-H···N and  N-H···N hydrogen bonding interactions. Inversion-related molecules form a dimer structure, in which two N—H···O hydrogen bonds generate an intermolecular *R*_2_^2^(8) ring (Table 1).

### Experimental

A mixture of the acetohydrazide (0.1 mol), and 1-(4-nitrophenyl)ethanone (0.1 mol) was stirred in refluxing ethanol (20 mL) for 4 h to afford the title compound (0.086 mol, yield 86%). Single crystals suitable for X-ray measurements were obtained by recrystallization from ethanol at room temperature.

### Refinement

N-bonded H atoms were found from a difference Fourier map and refined freely. C-bonded H atoms were fixed geometrically and allowed to ride on their attached atoms, with C—H = 0.93-0.96 Å, and with *U*_iso_=1.2–1.5*U*_eq_.

### Crystal data

**Table:**

C_10_H_11_N_3_O_3_	*Z* = 4
*M**_r_* = 221.22	*F*(000) = 464
Triclinic, *P*1	*D*_x_ = 1.347 Mg m^−^^3^
Hall symbol: -P 1	Mo *K*α radiation, λ = 0.71073 Å
*a* = 8.4453 (17) Å	Cell parameters from 1553 reflections
*b* = 9.5438 (19) Å	θ = 2.9–23.0°
*c* = 14.820 (3) Å	µ = 0.10 mm^−^^1^
α = 72.66 (3)°	*T* = 293 K
β = 77.37 (3)°	Block, yellow
γ = 75.59 (3)°	0.25 × 0.20 × 0.18 mm
*V* = 1090.7 (4) Å^3^	

### Data collection

**Table:**

Bruker SMART CCD area-detector diffractometer	2570 reflections with *I* > 2σ(*I*)
Radiation source: fine-focus sealed tube	*R*_int_ = 0.027
graphite	θ_max_ = 25.5°, θ_min_ = 2.3°
φ and ω scans	*h* = −10→10
6035 measured reflections	*k* = −8→11
4037 independent reflections	*l* = −17→17

### Refinement

**Table:**

Refinement on *F*^2^	Primary atom site location: structure-invariant direct methods
Least-squares matrix: full	Secondary atom site location: difference Fourier map
*R*[*F*^2^ > 2σ(*F*^2^)] = 0.052	Hydrogen site location: inferred from neighbouring sites
*wR*(*F*^2^) = 0.159	H atoms treated by a mixture of independent and constrained refinement
*S* = 1.04	*w* = 1/[σ^2^(*F*_o_^2^) + (0.0784*P*)^2^ + 0.1468*P*] where *P* = (*F*_o_^2^ + 2*F*_c_^2^)/3
4037 reflections	(Δ/σ)_max_ < 0.001
297 parameters	Δρ_max_ = 0.22 e Å^−^^3^
0 restraints	Δρ_min_ = −0.21 e Å^−^^3^

### Special details

**Table:**

Geometry. All e.s.d.'s (except the e.s.d. in the dihedral angle between two l.s. planes) are estimated using the full covariance matrix. The cell e.s.d.'s are taken into account individually in the estimation of e.s.d.'s in distances, angles and torsion angles; correlations between e.s.d.'s in cell parameters are only used when they are defined by crystal symmetry. An approximate (isotropic) treatment of cell e.s.d.'s is used for estimating e.s.d.'s involving l.s. planes.
Refinement. Refinement of *F*^2^ against ALL reflections. The weighted *R*-factor *wR* and goodness of fit *S* are based on *F*^2^, conventional *R*-factors *R* are based on *F*, with *F* set to zero for negative *F*^2^. The threshold expression of *F*^2^ > σ(*F*^2^) is used only for calculating *R*-factors(gt) *etc*. and is not relevant to the choice of reflections for refinement. *R*-factors based on *F*^2^ are statistically about twice as large as those based on *F*, and *R*- factors based on ALL data will be even larger.

### Fractional atomic coordinates and isotropic or equivalent isotropic displacement parameters (Å^2^)

**Table:**

	*x*	*y*	*z*	*U*_iso_*/*U*_eq_	
N5	0.6357 (2)	0.18317 (19)	0.43057 (12)	0.0478 (4)	
N4	0.6040 (2)	0.3087 (2)	0.46328 (13)	0.0517 (5)	
C20	0.7190 (3)	−0.0804 (3)	0.37732 (16)	0.0553 (6)	
H20A	0.7913	−0.0577	0.4076	0.066*	
O4	0.6882 (2)	0.4455 (2)	0.53613 (14)	0.0762 (6)	
C13	0.5290 (2)	0.1642 (2)	0.38741 (14)	0.0463 (5)	
C15	0.5727 (3)	0.0223 (2)	0.35793 (14)	0.0458 (5)	
C18	0.6510 (3)	−0.2475 (3)	0.30826 (16)	0.0540 (6)	
C19	0.7587 (3)	−0.2144 (3)	0.35260 (17)	0.0585 (6)	
H19A	0.8567	−0.2814	0.3657	0.070*	
C16	0.4685 (3)	−0.0151 (3)	0.31179 (16)	0.0574 (6)	
H16A	0.3708	0.0516	0.2975	0.069*	
N6	0.6913 (3)	−0.3924 (2)	0.28399 (15)	0.0688 (6)	
C12	0.7182 (3)	0.3345 (3)	0.50456 (18)	0.0597 (6)	
O6	0.5902 (3)	−0.4215 (2)	0.24812 (16)	0.0985 (7)	
O5	0.8220 (3)	−0.4769 (2)	0.30177 (14)	0.0845 (6)	
C14	0.3713 (3)	0.2712 (3)	0.36615 (19)	0.0659 (7)	
H14A	0.3614	0.3568	0.3898	0.099*	
H14B	0.2794	0.2229	0.3967	0.099*	
H14C	0.3719	0.3028	0.2982	0.099*	
C17	0.5074 (3)	−0.1501 (3)	0.28661 (17)	0.0629 (6)	
H17A	0.4370	−0.1738	0.2556	0.076*	
N2	0.3467 (2)	0.3952 (2)	0.09154 (13)	0.0517 (5)	
O1	−0.0396 (2)	0.6256 (2)	0.07168 (13)	0.0729 (5)	
N1	0.1929 (2)	0.4531 (2)	0.06573 (15)	0.0559 (5)	
C10	0.6576 (3)	0.3176 (2)	0.14039 (15)	0.0505 (5)	
H10A	0.5893	0.4061	0.1507	0.061*	
C3	0.4436 (3)	0.2916 (2)	0.05675 (15)	0.0497 (5)	
C8	0.9053 (3)	0.1382 (3)	0.15882 (16)	0.0537 (6)	
C5	0.6075 (3)	0.2378 (2)	0.09008 (15)	0.0468 (5)	
C6	0.7135 (3)	0.1075 (3)	0.07434 (17)	0.0601 (6)	
H6A	0.6834	0.0534	0.0402	0.072*	
C9	0.8052 (3)	0.2683 (2)	0.17494 (16)	0.0532 (6)	
H9A	0.8369	0.3222	0.2087	0.064*	
C7	0.8626 (3)	0.0568 (3)	0.10851 (18)	0.0640 (7)	
H7A	0.9327	−0.0307	0.0977	0.077*	
C2	0.0951 (3)	0.5678 (3)	0.09904 (17)	0.0555 (6)	
N3	1.0610 (3)	0.0837 (3)	0.19712 (18)	0.0759 (6)	
C1	0.1551 (3)	0.6207 (3)	0.16819 (19)	0.0722 (7)	
H1B	0.0741	0.7025	0.1856	0.108*	
H1C	0.2574	0.6534	0.1390	0.108*	
H1D	0.1724	0.5402	0.2244	0.108*	
C4	0.4032 (3)	0.2229 (3)	−0.0121 (2)	0.0814 (9)	
H4B	0.2938	0.2695	−0.0264	0.122*	
H4C	0.4084	0.1176	0.0160	0.122*	
H4D	0.4816	0.2376	−0.0701	0.122*	
O3	1.0875 (3)	0.1457 (3)	0.2518 (2)	0.1159 (9)	
O2	1.1593 (3)	−0.0208 (3)	0.1728 (2)	0.1231 (10)	
C11	0.8785 (3)	0.2256 (3)	0.5105 (3)	0.0922 (10)	
H11A	0.9474	0.2574	0.5410	0.138*	
H11B	0.9331	0.2208	0.4471	0.138*	
H11C	0.8582	0.1285	0.5470	0.138*	
H4A	0.513 (3)	0.378 (3)	0.4600 (17)	0.071 (8)*	
H1A	0.162 (3)	0.426 (3)	0.0248 (17)	0.054 (7)*	

### Atomic displacement parameters (Å^2^)

**Table:**

	*U*^11^	*U*^22^	*U*^33^	*U*^12^	*U*^13^	*U*^23^
N5	0.0487 (10)	0.0441 (10)	0.0552 (10)	−0.0028 (8)	−0.0128 (8)	−0.0215 (8)
N4	0.0477 (11)	0.0475 (11)	0.0665 (12)	0.0030 (9)	−0.0180 (9)	−0.0281 (9)
C20	0.0533 (13)	0.0543 (14)	0.0685 (15)	−0.0050 (11)	−0.0214 (11)	−0.0269 (12)
O4	0.0687 (11)	0.0712 (12)	0.1096 (14)	0.0064 (9)	−0.0348 (10)	−0.0562 (11)
C13	0.0445 (11)	0.0492 (13)	0.0455 (11)	−0.0056 (10)	−0.0105 (9)	−0.0132 (10)
C15	0.0492 (12)	0.0451 (12)	0.0441 (11)	−0.0079 (10)	−0.0100 (9)	−0.0122 (9)
C18	0.0669 (15)	0.0476 (13)	0.0536 (13)	−0.0128 (11)	−0.0091 (11)	−0.0209 (10)
C19	0.0583 (14)	0.0495 (14)	0.0731 (15)	−0.0017 (11)	−0.0171 (12)	−0.0262 (12)
C16	0.0570 (13)	0.0577 (14)	0.0645 (14)	−0.0059 (11)	−0.0239 (11)	−0.0204 (12)
N6	0.0906 (17)	0.0570 (14)	0.0680 (13)	−0.0219 (13)	−0.0070 (12)	−0.0275 (11)
C12	0.0513 (13)	0.0621 (15)	0.0749 (16)	0.0020 (11)	−0.0226 (12)	−0.0328 (13)
O6	0.1239 (17)	0.0867 (15)	0.1170 (17)	−0.0298 (13)	−0.0324 (14)	−0.0567 (13)
O5	0.0999 (16)	0.0583 (12)	0.0976 (15)	−0.0030 (11)	−0.0112 (12)	−0.0367 (11)
C14	0.0587 (15)	0.0656 (16)	0.0808 (17)	0.0078 (12)	−0.0275 (13)	−0.0345 (13)
C17	0.0732 (16)	0.0644 (16)	0.0653 (15)	−0.0189 (13)	−0.0224 (13)	−0.0250 (13)
N2	0.0469 (10)	0.0518 (11)	0.0593 (11)	−0.0039 (9)	−0.0174 (9)	−0.0169 (9)
O1	0.0565 (10)	0.0775 (12)	0.0890 (12)	0.0072 (9)	−0.0307 (9)	−0.0306 (10)
N1	0.0523 (11)	0.0576 (12)	0.0642 (12)	−0.0025 (9)	−0.0227 (10)	−0.0222 (10)
C10	0.0500 (13)	0.0439 (12)	0.0622 (13)	−0.0045 (10)	−0.0143 (10)	−0.0204 (10)
C3	0.0524 (13)	0.0453 (13)	0.0557 (13)	−0.0062 (10)	−0.0169 (10)	−0.0164 (10)
C8	0.0481 (12)	0.0546 (14)	0.0582 (13)	−0.0047 (11)	−0.0146 (10)	−0.0141 (11)
C5	0.0485 (12)	0.0460 (12)	0.0483 (12)	−0.0071 (10)	−0.0115 (9)	−0.0148 (10)
C6	0.0635 (15)	0.0550 (14)	0.0718 (15)	0.0016 (12)	−0.0231 (12)	−0.0330 (12)
C9	0.0518 (13)	0.0522 (14)	0.0634 (14)	−0.0092 (11)	−0.0140 (11)	−0.0237 (11)
C7	0.0594 (15)	0.0549 (15)	0.0794 (17)	0.0070 (12)	−0.0170 (12)	−0.0305 (13)
C2	0.0506 (13)	0.0542 (14)	0.0624 (14)	−0.0043 (11)	−0.0173 (11)	−0.0148 (11)
N3	0.0552 (13)	0.0743 (16)	0.1008 (18)	0.0038 (12)	−0.0305 (12)	−0.0271 (13)
C1	0.0635 (15)	0.0743 (18)	0.0880 (19)	0.0043 (13)	−0.0270 (14)	−0.0383 (15)
C4	0.0746 (18)	0.087 (2)	0.105 (2)	0.0091 (15)	−0.0418 (16)	−0.0579 (18)
O3	0.0844 (15)	0.132 (2)	0.159 (2)	0.0139 (14)	−0.0718 (15)	−0.0699 (18)
O2	0.0765 (14)	0.1116 (19)	0.196 (3)	0.0364 (14)	−0.0630 (16)	−0.0769 (19)
C11	0.0635 (17)	0.089 (2)	0.148 (3)	0.0211 (15)	−0.0522 (18)	−0.070 (2)

### Geometric parameters (Å, °)

**Table:**

N5—C13	1.287 (3)	N1—C2	1.349 (3)
N5—N4	1.368 (2)	N1—H1A	0.83 (2)
N4—C12	1.353 (3)	C10—C9	1.372 (3)
N4—H4A	0.88 (3)	C10—C5	1.397 (3)
C20—C19	1.376 (3)	C10—H10A	0.9300
C20—C15	1.398 (3)	C3—C5	1.489 (3)
C20—H20A	0.9300	C3—C4	1.500 (3)
O4—C12	1.231 (3)	C8—C9	1.371 (3)
C13—C15	1.481 (3)	C8—C7	1.376 (3)
C13—C14	1.493 (3)	C8—N3	1.460 (3)
C15—C16	1.391 (3)	C5—C6	1.389 (3)
C18—C17	1.367 (3)	C6—C7	1.380 (3)
C18—C19	1.374 (3)	C6—H6A	0.9300
C18—N6	1.472 (3)	C9—H9A	0.9300
C19—H19A	0.9300	C7—H7A	0.9300
C16—C17	1.389 (3)	C2—C1	1.492 (3)
C16—H16A	0.9300	N3—O2	1.218 (3)
N6—O6	1.222 (3)	N3—O3	1.219 (3)
N6—O5	1.223 (3)	C1—H1B	0.9600
C12—C11	1.489 (3)	C1—H1C	0.9600
C14—H14A	0.9600	C1—H1D	0.9600
C14—H14B	0.9600	C4—H4B	0.9600
C14—H14C	0.9600	C4—H4C	0.9600
C17—H17A	0.9300	C4—H4D	0.9600
N2—C3	1.277 (3)	C11—H11A	0.9600
N2—N1	1.368 (3)	C11—H11B	0.9600
O1—C2	1.232 (3)	C11—H11C	0.9600
			
C13—N5—N4	119.06 (18)	C5—C10—H10A	119.3
C12—N4—N5	119.98 (19)	N2—C3—C5	115.09 (19)
C12—N4—H4A	114.2 (17)	N2—C3—C4	125.2 (2)
N5—N4—H4A	125.8 (17)	C5—C3—C4	119.7 (2)
C19—C20—C15	121.5 (2)	C9—C8—C7	122.0 (2)
C19—C20—H20A	119.2	C9—C8—N3	118.9 (2)
C15—C20—H20A	119.2	C7—C8—N3	119.1 (2)
N5—C13—C15	114.65 (18)	C6—C5—C10	117.9 (2)
N5—C13—C14	125.5 (2)	C6—C5—C3	122.1 (2)
C15—C13—C14	119.81 (19)	C10—C5—C3	120.00 (19)
C16—C15—C20	117.6 (2)	C7—C6—C5	121.2 (2)
C16—C15—C13	121.3 (2)	C7—C6—H6A	119.4
C20—C15—C13	121.07 (18)	C5—C6—H6A	119.4
C17—C18—C19	121.9 (2)	C8—C9—C10	118.8 (2)
C17—C18—N6	119.2 (2)	C8—C9—H9A	120.6
C19—C18—N6	118.9 (2)	C10—C9—H9A	120.6
C18—C19—C20	118.9 (2)	C8—C7—C6	118.7 (2)
C18—C19—H19A	120.6	C8—C7—H7A	120.7
C20—C19—H19A	120.6	C6—C7—H7A	120.7
C17—C16—C15	121.3 (2)	O1—C2—N1	119.6 (2)
C17—C16—H16A	119.4	O1—C2—C1	122.2 (2)
C15—C16—H16A	119.4	N1—C2—C1	118.3 (2)
O6—N6—O5	123.9 (2)	O2—N3—O3	122.8 (2)
O6—N6—C18	117.5 (2)	O2—N3—C8	118.8 (2)
O5—N6—C18	118.5 (2)	O3—N3—C8	118.4 (2)
O4—C12—N4	120.0 (2)	C2—C1—H1B	109.5
O4—C12—C11	121.8 (2)	C2—C1—H1C	109.5
N4—C12—C11	118.2 (2)	H1B—C1—H1C	109.5
C13—C14—H14A	109.5	C2—C1—H1D	109.5
C13—C14—H14B	109.5	H1B—C1—H1D	109.5
H14A—C14—H14B	109.5	H1C—C1—H1D	109.5
C13—C14—H14C	109.5	C3—C4—H4B	109.5
H14A—C14—H14C	109.5	C3—C4—H4C	109.5
H14B—C14—H14C	109.5	H4B—C4—H4C	109.5
C18—C17—C16	118.8 (2)	C3—C4—H4D	109.5
C18—C17—H17A	120.6	H4B—C4—H4D	109.5
C16—C17—H17A	120.6	H4C—C4—H4D	109.5
C3—N2—N1	120.00 (19)	C12—C11—H11A	109.5
C2—N1—N2	119.4 (2)	C12—C11—H11B	109.5
C2—N1—H1A	118.7 (16)	H11A—C11—H11B	109.5
N2—N1—H1A	121.5 (16)	C12—C11—H11C	109.5
C9—C10—C5	121.4 (2)	H11A—C11—H11C	109.5
C9—C10—H10A	119.3	H11B—C11—H11C	109.5

### Hydrogen-bond geometry (Å, °)

**Table:**

*D*—H···*A*	*D*—H	H···*A*	*D*···*A*	*D*—H···*A*
N1—H1A···O1^i^	0.84 (3)	2.16 (3)	2.989 (3)	170 (2)
N4—H4A···O4^ii^	0.88 (3)	2.08 (3)	2.952 (3)	172 (2)
C4—H4B···N1	0.96	2.42	2.826 (3)	105
C4—H4B···O1^i^	0.96	2.28	3.243 (3)	175
C10—H10A···O6^iii^	0.93	2.49	3.216 (3)	135
C14—H14A···N4	0.96	2.41	2.821 (3)	105
C14—H14A···O4^ii^	0.96	2.36	3.317 (3)	173
C16—H16A···O3^iv^	0.93	2.50	3.388 (4)	159
C20—H20A···N5	0.93	2.43	2.740 (3)	100

Symmetry codes: (i) −*x*, −*y*+1, −*z*; (ii) −*x*+1, −*y*+1, −*z*+1; (iii) *x*, *y*+1, *z*; (iv) *x*−1, *y*, *z*.
